# Epidural Abscess in Routine Preoperative MRI Reports Versus Documented Operative Findings, and Exploratory Mortality Risk Factors in Surgically Treated Spondylodiscitis: A Retrospective Single-Centre Study

**DOI:** 10.3390/jcm15197665

**Published:** 2026-10-03

**Authors:** Bahadır Balkanlı, Enver İpek, Mehmet Alp Yüksel, Oğuzhan Nayman, Yusuf Sülek, Mehmet Ali Talmaç

**Affiliations:** 1 Department of Orthopaedics and Traumatology, Şişli Hamidiye Etfal Training and Research Hospital, University of Health Sciences, 34396 Istanbul, Türkiye; 2 Department of Orthopaedics and Traumatology, Esenyurt Necmi Kadıoğlu State Hospital, Esenyurt, 34517 Istanbul, Türkiye; 3 Department of Orthopaedics and Traumatology, Başakşehir Çam ve Sakura City Hospital, University of Health Sciences, Başakşehir, 34480 Istanbul, Türkiye

**Keywords:** spondylodiscitis, epidural abscess, magnetic resonance imaging, radiology report, Charlson comorbidity index, mortality

## Abstract

**Background:** Surgical decisions in spondylodiscitis rely on the preoperative magnetic resonance imaging (MRI) report, and postoperative mortality remains high. We examined how often routine MRI reports described an epidural abscess later documented at surgery and explored factors associated with mortality. **Methods:** Twenty-seven adults operated on for pyogenic or fungal spondylodiscitis at one centre (2017–2025) were studied retrospectively. Findings were abstracted from routine MRI reports and operative notes, with epidural pus documented at surgery as the reference. Mortality was explored with univariable Cox and receiver operating characteristic analyses. **Results:** Epidural pus was documented in 11 patients; none of their MRI reports described an epidural abscess (sensitivity 0%, 95% confidence interval [CI] 0–28.5%), although six reported paraspinal soft-tissue involvement. Eleven patients (40.7%) died during a median follow-up of 2.5 months. Age (hazard ratio 1.14 per year, 95% CI 1.00–1.30) and the age-adjusted Charlson Comorbidity Index (1.54 per point, 1.05–2.28) were associated with mortality and discriminated similarly (areas under the curve 0.741 and 0.736); the unadjusted index and the Pola, Homagk, Spinal Instability Neoplastic Score, and Spinal Instability Spondylodiscitis Score classifications were not. **Conclusions:** In this small retrospective cohort, routine MRI reports named no epidural abscess among patients in whom one was documented at surgery. Mortality was associated with age and age-adjusted comorbidity burden. These exploratory findings require prospective validation.

## 1. Introduction

Spondylodiscitis is an uncommon but demanding infection of the intervertebral disc and adjacent vertebral endplates. Its incidence in high-income settings is roughly five to six per hundred thousand person-years and has risen steadily over two decades [1,2], driven by an ageing population and the wider use of implantable devices, vascular catheters, and immunomodulatory therapy [3,4]. Most cases can be controlled with targeted antimicrobial therapy and orthotic support, but a substantial minority come to surgery for instability, structural collapse, neurological deficit, epidural or paravertebral abscess, or failure of conservative treatment [5,6,7].

Even in specialist centres, mortality after surgery remains high—commonly fifteen to thirty per cent [8,9,10]. Early surgery may lower mortality relative to conservative management [11], yet outcomes within surgical series are far from uniform and turn on the interplay of age, comorbidity burden, and severity of local disease [12,13,14]. Identifying which patients are at greatest risk, at the moment of surgical decision-making, is therefore central to current practice.

Several systems have been developed to characterise spondylodiscitis: the Pola classification, built on segmental instability and neurological involvement [15,16]; the Homagk score (SponDT), grading severity across five types [17]; the Spinal Instability Neoplastic Score (SINS), devised for metastatic disease but increasingly applied to infection [18]; and the Spinal Instability Spondylodiscitis Score (SISS), a disease-specific counterpart of SINS [19,20,21]. All were intended chiefly to guide treatment intensity, and their behaviour in anticipating death has been examined only inconsistently [22,23,24,25,26]. In parallel, generic comorbidity indices—above all the Charlson Comorbidity Index and its age-adjusted variant—have shown robust associations with mortality across spinal surgical populations, including elective fusion, spinal trauma, and epidural abscess [27,28,29,30,31]. How these generic indices and the disease-specific classifications relate to mortality has rarely been examined within the same surgical cohort.

A separate concern is that surgical decision-making leans heavily on preoperative magnetic resonance imaging (MRI), used both to establish the diagnosis and to characterise local disease [32]. How closely the routine MRI report reflects what is later found at operation—for epidural abscess in particular—has received little scrutiny, even though surgeons not uncommonly find at operation collections unanticipated on imaging [33,34]. Notably, the most sensitive sign of epidural abscess on unenhanced MRI is not the collection itself but the adjacent paraspinal oedema [33], raising the possibility that an abscess may be represented indirectly, through soft-tissue signal, even when not named as such in the routine report.

In this study, we set out to address these gaps within a single surgical cohort of adult patients with pyogenic or fungal spondylodiscitis, treated over a nine-year period at one tertiary centre. Specifically, we aimed (i) to describe how often routine preoperative MRI reports described an epidural abscess that was subsequently documented at surgery; and (ii), as a secondary and exploratory aim, to examine the association of age, comorbidity indices, a composite MRI severity score, and four classification systems with all-cause mortality. Tuberculous cases were excluded a priori, since their distinct natural history and prognostic profile would confound the intended comparison [6].

## 2. Materials and Methods

### 2.1. Study Design and Setting

We conducted a single-centre retrospective cohort study in the Department of Orthopaedics and Traumatology of a tertiary referral hospital, covering patients treated between 1 January 2017 and 31 December 2025. The department is a regional referral centre for complex spinal infections, and all operations were performed by the same senior spine surgery team with a consistent approach throughout.

The study is reported in line with the Standards for Reporting of Diagnostic Accuracy Studies (STARD) guideline for its diagnostic-accuracy component and the Strengthening the Reporting of Observational Studies in Epidemiology (STROBE) guideline for its observational design. We hypothesised that routine preoperative MRI reports would describe epidural abscess less often than it was documented at surgery, and that morphology-based classifications, which were not designed to predict mortality, would show weaker associations with mortality than age-adjusted comorbidity burden. The mortality analyses were exploratory.

### 2.2. Cohort Definition and Eligibility

Consecutive adults (≥18 years) who underwent surgery for spondylodiscitis were screened. The diagnosis rested on the characteristic clinical picture—intractable back pain and raised inflammatory markers, with or without neurological signs—together with MRI changes involving the disc and adjacent endplates, consistent with the IDSA criteria of Berbari and colleagues [5]. Cases were confirmed microbiologically by positive tissue or blood culture, or, in culture-negative patients, clinically by imaging findings and treatment response.

Patients were included if they had (i) pyogenic or fungal spondylodiscitis, (ii) surgical treatment (debridement alone or with instrumentation), and (iii) at least one month of follow-up or in-hospital death within that interval.

Patients with tuberculous spondylodiscitis (Pott disease) were excluded a priori: tuberculous disease follows an indolent chronic course, requires prolonged multidrug therapy, and carries a prognostic profile not comparable with pyogenic infection, so that combining the two would have obscured the mortality signal and limited the interpretability of classifications not developed for tuberculosis. Fungal cases were retained, since fungal spondylodiscitis resembles pyogenic disease in its acuity, abscess formation, and treatment principles.

**Figure 1:**
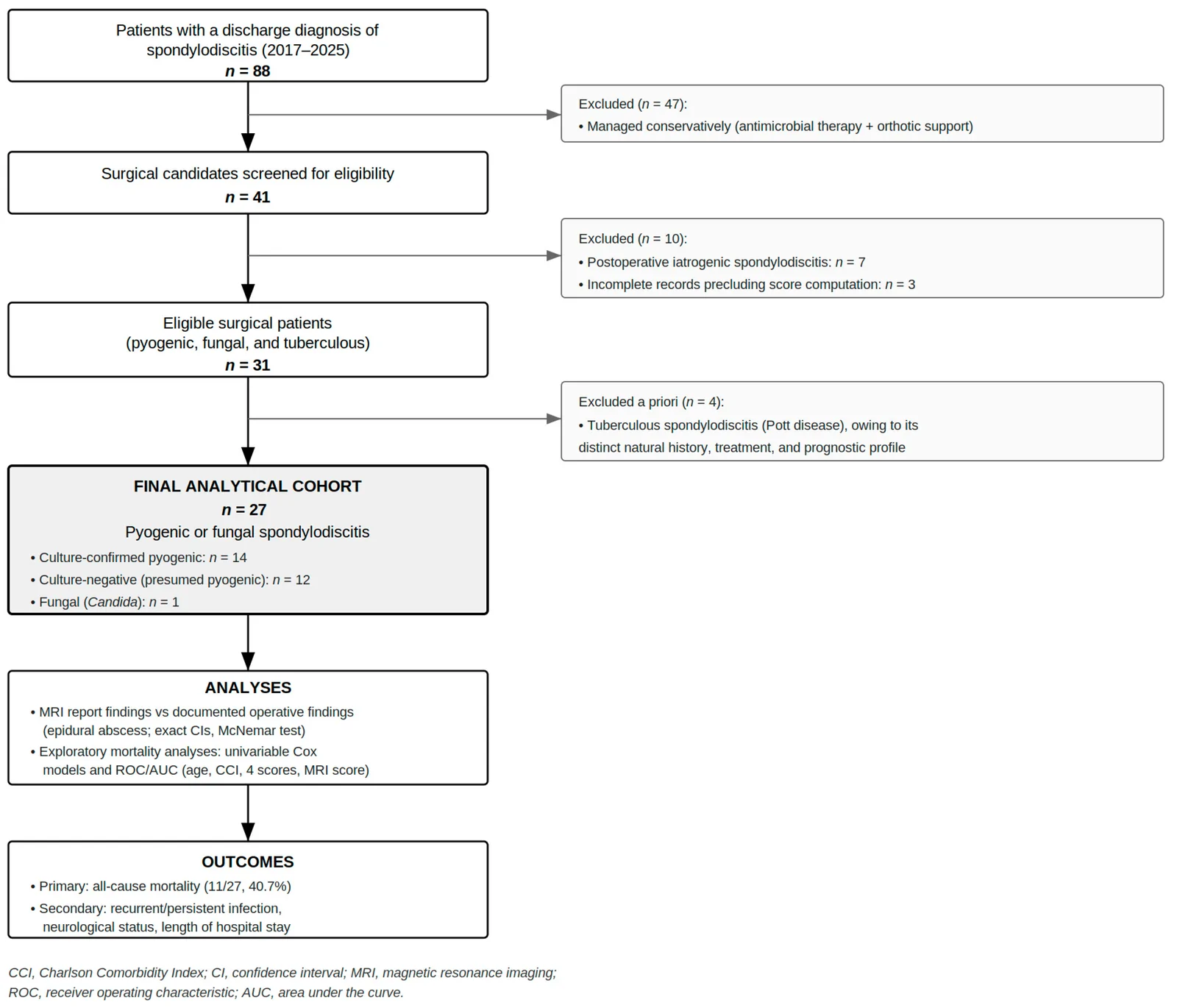
STROBE flow diagram of patient selection and analyses. Of 88 patients with a discharge diagnosis of spondylodiscitis (2017–2025), 47 were managed conservatively and 10 were excluded (7 postoperative iatrogenic; 3 incomplete records); after a priori exclusion of 4 patients with tuberculous spondylodiscitis, 27 patients with pyogenic or fungal spondylodiscitis formed the final cohort.

Further exclusion criteria were postoperative spondylodiscitis after elective spinal surgery (a distinct entity), age below 18 years, and incomplete records precluding score computation. Of the eligible patients, four were excluded for confirmed tuberculosis, leaving a final cohort of twenty-seven (Figure 1).

### 2.3. Clinical Assessment and Scoring Systems

Demographic and clinical data—age, sex, American Society of Anesthesiologists (ASA) physical status, presenting symptoms, the presence and duration of neurological deficit, and comorbid conditions—were extracted independently by two investigators, with discrepancies resolved by consensus. Laboratory parameters were recorded preoperatively and on postoperative day 7, chosen to capture the early postoperative trend, and comprised white blood cell count, haemoglobin, C-reactive protein, erythrocyte sedimentation rate, and serum albumin.

Comorbidity burden was quantified with the Charlson Comorbidity Index (CCI) as defined by Charlson and colleagues [27,28] and its age-adjusted variant, which adds one point per decade of age beyond forty years.

Four classification systems were assigned to each patient by consensus of two spine surgeons blinded to outcome; individual pre-consensus ratings were not recorded. The systems were the Pola classification (categories A–C by segmental instability and neurological involvement); the Homagk classification (SponDT, types 1–5); the Spinal Instability Neoplastic Score (SINS), developed for metastatic disease but also applied to infection; and the Spinal Instability Spondylodiscitis Score (SISS), a disease-specific analogue of SINS.

### 2.4. Radiological Assessment

All patients underwent preoperative MRI on a 1.5-Tesla scanner (Signa, GE Medical Systems, Milwaukee, WI, USA, in 2017–2019; MAGNETOM Sola, Siemens Healthineers, Erlangen, Germany, from 2020 onwards) using the standard institutional protocol for suspected spinal infection: sagittal T1, T2, and short tau inversion recovery (STIR) sequences, axial T2 images at the affected level, and contrast-enhanced T1 after intravenous gadolinium; contrast-enhanced sequences were obtained in all 27 patients. MRI was performed 1–7 days before surgery and the operation was planned on this most recent examination; the exact interval was not recorded for each patient.

Preoperative MRI findings were coded in binary form (present or absent) from the formal radiology reports prepared by attending musculoskeletal radiologists during routine care. The morphological findings recorded were epidural abscess, paravertebral abscess, cord compression, intramedullary signal change, vertebral body destruction, endplate involvement, and paraspinal soft-tissue involvement. The first six were summed into a composite MRI severity score from zero (no abnormality beyond disc–endplate infection) to six (all present); this score was constructed for exploratory purposes and has not been validated. Paraspinal soft-tissue involvement was recorded separately and not included in the score.

MRI examinations were not re-read for this study; findings were taken from the wording of the routine clinical reports. The analysis therefore describes the performance of routine reporting, as available to the surgeon at the time of decision-making, and not the diagnostic accuracy of MRI itself.

Operative findings were likewise extracted retrospectively from the operative notes, which had been written for clinical rather than research purposes, using the same binary categories. An epidural abscess was recorded as present when purulent material in the epidural space was documented in the operative note; purulent material alone constituted the reference standard. The epidural space was inspected in every patient: by direct exploration and decompression in the six patients whose MRI report described an epidural abscess or cord compression, and through the debridement corridor (anterior corpectomy or discectomy, or posterior debridement) in the remaining 21. Because the extent of exposure differed between these approaches, the documented operative finding cannot be regarded as an error-free reference.

### 2.5. Surgical Treatment

Surgical indications followed current practice and included one or more of: progressive or fixed neurological deficit from compression; segmental instability or destructive change compromising anterior column integrity; failure of conservative treatment (clinical or biochemical persistence despite adequate antimicrobial therapy); and the need for tissue sampling in microbiologically undefined patients. The approach was individualised to the level and extent of destruction: posterior in 18 patients, anterior in 3, and combined anterior–posterior in 6. In all cases, the sequence comprised radical debridement, decompression of any epidural collection, and anterior column reconstruction with autologous graft, a titanium cage, or both; pedicle-screw instrumentation was added when instability was demonstrated intraoperatively or the extent of vertebrectomy required it.

Antimicrobial therapy was managed by infectious diseases specialists. Because no patient had sepsis, haemodynamic instability, or a rapidly progressive neurological deficit, antibiotics were not started before surgery, in order to maximise the yield of intraoperative cultures [5]. Postoperatively, pathogen-directed therapy was given when an organism was isolated and empirical therapy otherwise, with a planned total duration of 6–12 weeks; individual intravenous and total treatment durations were not recorded consistently and are therefore not reported.

### 2.6. Follow-Up and Outcome Definitions

Patients were followed in the outpatient clinic after discharge according to a standard protocol, at 2, 4, 6, and 12 weeks after surgery and every three months thereafter during the first year. Follow-up was calculated in months from surgery to the last documented contact or death. The primary outcome was all-cause mortality during follow-up, ascertained from the medical record and, where necessary, the national civil registry; both in-hospital and post-discharge deaths were counted. No distinction was made between infection-related and non-infection-related death, as such attribution is unreliable here.

Secondary outcomes were recurrent or persistent infection, neurological status at the end of the orthopaedic ward stay, and length of hospital stay. Neurological status was graded with the American Spinal Injury Association (ASIA) Impairment Scale on admission and at the end of the orthopaedic ward stay, as recorded in the orthopaedic discharge or transfer summary. In patients with a deficit at presentation, the change was categorised as complete recovery, partial improvement, or no change; in all patients, the development of a new deficit was recorded, and patients who could not be examined reliably were classified as not assessable. Neurological status after transfer to other wards was not captured. Length of stay was recorded separately for the orthopaedic ward, the intensive care unit, and the infectious diseases ward, and summed to give the total length of the index admission.

### 2.7. Statistical Analysis

Continuous variables were summarised as median (interquartile range, IQR) and categorical variables as frequency and percentage. Survivors and non-survivors were compared with the Mann–Whitney U test for continuous variables and the chi-square or Fisher exact test for categorical variables; paired laboratory values were compared with the Wilcoxon signed-rank test.

For epidural abscess, the MRI report finding was compared with the documented operative finding. Sensitivity, specificity, and predictive values were calculated with exact (Clopper–Pearson) 95% confidence intervals (CIs). Directional discordance between the two sources was assessed with the exact McNemar test, and a non-significant result was not interpreted as evidence of agreement. The association between reported paraspinal soft-tissue involvement and documented epidural pus was assessed with the Fisher exact test and summarised as a conditional maximum-likelihood odds ratio with an exact 95% CI.

Because follow-up varied widely (0.3–32.5 months) and a fixed mortality horizon could not be applied without excluding patients censored early, the association of each measure with all-cause mortality was examined primarily with univariable Cox proportional hazards models, using follow-up time from surgery and death as the event. The proportional hazards assumption was checked with tests based on scaled Schoenfeld residuals, and discrimination was summarised with Harrell’s concordance index. As a secondary, descriptive analysis, discrimination for death during follow-up was expressed as the area under the receiver operating characteristic (ROC) curve (AUC) with DeLong 95% CIs, and AUCs were compared pairwise with the DeLong test [35]; age alone was analysed alongside the unadjusted and age-adjusted CCI. A cut-off for the age-adjusted CCI was derived with the Youden index and survival above and below it was compared with the log-rank test; because this threshold was derived and evaluated in the same small cohort, it was regarded as hypothesis-generating only.

Given the eleven deaths, and in keeping with the rule of at least ten events per variable, no multivariable model was fitted; the separate contributions of age and comorbidity could therefore not be disentangled, and all mortality analyses were considered exploratory. For comparison with the original analysis, a univariable logistic model for death during follow-up was also fitted. Pairwise correlations between classifications were expressed as Spearman rank coefficients.

Analyses were performed with IBM SPSS Statistics version 27 (IBM Corp., Armonk, NY, USA) and Python 3.12 (Python Software Foundation, Wilmington, DE, USA; SciPy 1.17, scikit-learn 1.8, and lifelines 0.30); exact CIs, ROC analyses with DeLong CIs and comparisons, and Cox models were computed in Python. A two-sided *p* < 0.05 was taken as the threshold for statistical significance; no adjustment was made for multiple comparisons.

## 3. Results

### 3.1. Cohort Characteristics

The final cohort comprised twenty-seven patients (Table 1). Median age was 67 years (IQR 60–70; range 28–77) and most were men (20/27, 74.1%). The median ASA score was 3, and comorbidity burden was considerable: 85.2% (23/27) had at least one comorbid condition, most often cardiac (66.7%) and metabolic (33.3%). The median Charlson Comorbidity Index was 1 (IQR 0–2) and the median age-adjusted CCI was 3 (IQR 2–4).

All patients were admitted to the intensive care unit postoperatively, and 20 (74.1%) were subsequently transferred to the infectious diseases ward. The median total length of the index admission was 25 days (IQR 22–29.5; range 14–45), comprising a median of 8 days (IQR 4–13) on the orthopaedic ward, 4 days (IQR 2.5–5) in intensive care, and 14 days (IQR 3.5–18) on the infectious diseases ward. Median follow-up was 2.5 months (IQR 1.2–8.3; range 0.3–32.5), and seven of the 16 survivors were followed for less than three months. Eleven patients died during follow-up, giving an observed all-cause mortality of 40.7%.

### 3.2. Microbiological, Radiological, and Surgical Findings

Infection most often involved the lumbar or lumbosacral spine (15/27, 55.6%), followed by the thoracic (25.9%) and thoracolumbar (14.8%) regions. Tissue culture was positive in 55.6% of patients and blood culture in 48.1%. Microbiologically, fourteen patients had culture-positive pyogenic infection (most often methicillin-sensitive *Staphylococcus aureus*, 7 patients), twelve had culture-negative but clinically pyogenic disease, and one had a fungal (*Candida* species) infection (Table 2).

The findings most frequently reported were vertebral body destruction (33.3%), endplate involvement (29.6%), and paraspinal soft-tissue involvement (29.6%); by contrast, epidural abscess was reported in only two patients (7.4%). The median composite MRI severity score was 1 (IQR 0–2). The surgical approach was posterior in 18 patients (66.7%), anterior in 3 (11.1%), and combined in 6 (22.2%); the epidural space was explored directly in 6 patients and inspected through the debridement corridor in the remaining 21. Instrumentation was used in 25 patients (92.6%), spanning a median of 4 levels (IQR 3–6). Purulent material in the epidural space was documented in the operative notes of 11 patients (40.7%).

### 3.3. Epidural Abscess in MRI Reports and Operative Notes

Among the 11 patients with epidural pus documented at surgery, none had an epidural abscess described in the preoperative MRI report, giving a sensitivity of 0% for the reported label (0/11; exact 95% CI 0–28.5%) (Table 3, Panel A). Conversely, neither of the two patients whose report described an epidural abscess had epidural pus documented at surgery; one had a reported paravertebral abscess with cord compression and the other paraspinal soft-tissue involvement. Specificity was 87.5% (14/16; 95% CI 61.7–98.4%), and the discordance between the two sources was directional (exact McNemar *p* = 0.022).

Paraspinal soft-tissue involvement had been reported in 6 of the 11 patients with documented epidural pus (54.5%; 95% CI 23.4–83.3%) and in 2 of the 16 without (12.5%) (Fisher exact *p* = 0.033; conditional odds ratio 7.65, exact 95% CI 0.97–102.2). The wide confidence interval indicates that this association is estimated with considerable imprecision. The McNemar test did not show directional discordance between reported soft-tissue involvement and documented epidural pus (*p* = 0.45); given the small numbers, this should not be taken as evidence of agreement.

### 3.4. Perioperative Changes in Laboratory Parameters

Laboratory values on postoperative day 7 differed from preoperative values (Table 4). C-reactive protein decreased from a median of 83.5 to 26.0 mg/L (*p* = 0.005) and erythrocyte sedimentation rate from 66.0 to 32.0 mm/h (*p* = 0.001). White blood cell count was higher on day 7 than preoperatively (median 7.45 vs. 10.95 × 10^3^/µL; *p* = 0.001); this may reflect the postoperative inflammatory response, although persistent infection cannot be excluded. Serum albumin decreased (from 3.20 to 2.63 g/dL; *p* = 0.024), whereas the change in haemoglobin did not reach significance (*p* = 0.059).

### 3.5. Factors Associated with Mortality (Exploratory)

In univariable Cox models, age (hazard ratio [HR] 1.14 per year; 95% CI 1.00–1.30; *p* = 0.043) and the age-adjusted CCI (HR 1.54 per point; 95% CI 1.05–2.28; *p* = 0.029) were associated with mortality, whereas the unadjusted CCI was not (HR 1.45; 95% CI 0.93–2.26; *p* = 0.098) (Table 3, Panel C). Harrell’s concordance index was 0.713 for age, 0.724 for the age-adjusted CCI, and 0.665 for the unadjusted CCI, and the proportional hazards assumption was not violated for any measure. Age and the age-adjusted CCI were correlated (Spearman ρ = 0.62), and age differed between survivors and non-survivors (Table S2). Neither the four classification systems nor the composite MRI severity score were associated with mortality (all *p* ≥ 0.110); score distributions are given in Table S1.

On ROC analysis (Table 3, Panel B), the AUCs of age (0.741; 95% CI 0.544–0.939) and the age-adjusted CCI (0.736; 95% CI 0.553–0.918) were nearly identical (difference −0.006; DeLong *p* = 0.96), and the age-adjusted CCI did not differ significantly from the unadjusted CCI (*p* = 0.26). The AUCs of the classification systems and the MRI severity score ranged from 0.440 to 0.625, with 95% CIs including 0.50. Of the pairwise comparisons (Table S4), only that between the age-adjusted CCI and the Homagk classification was nominally significant (*p* = 0.036), and it would not remain so after correction for multiple comparisons. In a univariable logistic model that ignored follow-up time, the age-adjusted CCI was not significantly associated with death (odds ratio 1.71; 95% CI 0.98–2.96; *p* = 0.058). At the Youden-derived threshold (>3), median survival was 2.2 months in the higher group and 21.0 months in the lower group, but the difference did not reach significance (log-rank *p* = 0.055).

### 3.6. Neurological Course

Twelve patients (44.4%) presented with a neurological deficit, and all were examined at the end of the orthopaedic ward stay: the deficit had resolved in five, improved in five, and was unchanged in two. Six of these twelve patients died later during follow-up. Of the 15 patients without a deficit at presentation, 14 had no new deficit at that time and one could not be assessed.

## 4. Discussion

### 4.1. Principal Findings

In this retrospective surgical cohort, none of the 11 patients with epidural pus documented at operation had an epidural abscess described in the preoperative MRI report, although paraspinal soft-tissue involvement had been reported in six of them. In exploratory analyses, mortality was associated with age and with the age-adjusted CCI, which showed nearly identical discrimination, whereas comorbidity alone, the composite MRI severity score, and the four classification systems were not associated with death. Both sets of observations derive from retrospective clinical documentation in a small sample and should be regarded as hypothesis-generating.

### 4.2. Interpretation of the Discordance Between MRI Reports and Operative Notes

The infrequency with which an epidural abscess was named in routine reports is the main observation of this study, but it compares two clinical documents rather than MRI with an independent reference. The absence of the term in a report does not establish that a collection was not visible on the images, and the images were not re-read for this study. Several explanations are therefore possible. A collection may have been visible but described in other terms or not regarded as the principal finding; early phlegmonous change can be difficult to distinguish from a mature collection; and because MRI was obtained up to seven days before surgery in patients who had not yet received antibiotics, a collection may have formed or enlarged in the interval. Conversely, the operative notes were written for clinical purposes, and although the epidural space was inspected in every patient, the extent of exposure differed between direct exploration and the debridement corridor. In the two patients whose report described an epidural abscess, no epidural pus was documented at surgery, which may reflect phlegmon rather than pus or differences in exposure.

Paraspinal soft-tissue involvement was reported more often in patients with documented epidural pus (6/11) than in those without (2/16). Paraspinal oedema has been described as a sensitive, though non-specific, feature of epidural abscess on unenhanced MRI [33], so this pattern is biologically plausible. In our data, however, the association was estimated with wide uncertainty (exact 95% CI for the odds ratio 0.97–102.2), and it does not show that an unreported epidural abscess was identifiable on the original images. It therefore raises a question for future studies with blinded, protocol-based image review rather than providing an answer.

Timely decompression matters for patients with a neurological deficit from spinal epidural abscess [34], and the European Association of Neurosurgical Societies (EANS) consensus recommends that surgical planning assess epidural extension carefully rather than rely on a single dichotomous imaging statement [6]. Our observations are not an argument against MRI, which remains indispensable for diagnosis and planning. They suggest only that, in this setting, the absence of the term “epidural abscess” in a routine report should not be relied on to exclude an epidural collection, and that report wording may deserve attention in future work on structured reporting. We are not aware of previous surgical series of spondylodiscitis that compared routine MRI reports with documented operative findings for epidural abscess, but our data describe the reporting practice of a single centre and may not be generalisable.

### 4.3. Classification Systems and Mortality

None of the four classification systems was associated with mortality in this cohort. This should be read as an exploratory observation rather than a failure of the scores: Pola and Homagk were developed to guide treatment intensity [15,16,17], SINS to assess instability in metastatic disease [18], and SISS to grade instability in spondylodiscitis [19,20,21], and none was designed to predict death. The four scores were partly correlated with one another (Table S3), consistent with their capturing overlapping aspects of local disease. Their lack of association with mortality is in keeping with this intended scope and with reports that classification-derived severity relates to functional outcomes more than to mortality [23]; scores developed for treatment allocation may behave differently when used for outcome prediction [20,22], and the EANS consensus notes that no existing score has been validated for mortality in this setting [6]. With eleven deaths, our data cannot exclude modest associations, and dedicated prognostic instruments such as the Mortality in Spinal Infection (MSI-20) score [26] are a more appropriate route to mortality prediction.

### 4.4. Age, Comorbidity, and Mortality

The age-adjusted CCI differs in kind from the classification systems, because it incorporates age and comorbidity, both of which are directly related to mortality; its association with death is therefore not directly comparable with theirs. In our cohort, age alone and the age-adjusted CCI showed nearly identical discrimination; the two were correlated, and comorbidity without age was not significantly associated with mortality. Within a sample of this size the contribution of comorbidity cannot be separated from that of age, and we therefore do not interpret our findings as showing that comorbidity is the dominant determinant of mortality. The Youden-derived threshold of the age-adjusted CCI was derived and evaluated in the same small cohort and did not reach significance on the log-rank test; it should be regarded as a hypothesis for external testing rather than a threshold for counselling or perioperative management.

These observations are consistent with larger series in which multiorgan failure was the leading cause of in-hospital death [10] and age and comorbidity were among the most consistent predictors of mortality after surgery [13,14], and with the association between the CCI and mortality in other spinal surgical populations [29,30,31]. Whether a generic comorbidity measure adds prognostic information beyond age in surgically treated spondylodiscitis requires larger cohorts that allow multivariable modelling.

### 4.5. Interpretation of the Neurological Findings

All twelve patients with a neurological deficit at presentation were examined at the end of the orthopaedic ward stay; the deficit resolved or improved in ten, and no new deficit was recorded among the fourteen assessable patients without a deficit at presentation. Six of the twelve patients with a presenting deficit died later during follow-up, and neurological status after transfer to other wards was not recorded, so these figures describe short-term neurological status and do not allow conclusions about final neurological outcome or about the relationship between neurological recovery and survival.

### 4.6. Clinical Implications

Two cautious implications follow. First, in patients with clinical features of aggressive disease, the absence of an epidural abscess in the routine MRI report should not by itself be taken to exclude an epidural collection, and reviewing the images with the reporting radiologist before surgery may be worthwhile. Second, age and comorbidity burden should be considered alongside disease-specific classifications when discussing perioperative risk, recognising that the present data do not support a specific threshold for clinical decisions.

### 4.7. Strengths and Limitations

The study has some strengths: consecutive patients treated over nine years at a single centre by a consistent surgical team and reported by the same radiology service; a priori exclusion of tuberculous cases; classification scoring blinded to outcome; and inspection of the epidural space in every patient.

The limitations are considerable. The study is retrospective, single-centre, and small, which limits statistical power and generalisability. Both MRI and operative findings were abstracted from clinical documents written for care rather than research: the images were not re-read under a standardised protocol by blinded readers, so our findings describe routine reporting rather than the diagnostic accuracy of MRI, and the operative notes were not standardised, with the extent of epidural exposure differing between approaches. The interval between MRI and surgery was recorded only as a range of 1–7 days. Classification scores were assigned by consensus, and inter-rater reliability could not be assessed because individual ratings were not recorded. Individual durations of intravenous and total antimicrobial therapy were not reliably available, and neurological status was recorded at the end of the orthopaedic ward stay rather than at final hospital discharge. With eleven deaths, no multivariable model could be fitted, so the independent contributions of age and comorbidity could not be separated; the Youden threshold was derived and tested in the same cohort; and several comparisons were made without adjustment for multiplicity. Follow-up was short for many survivors, and cause of death was not adjudicated, so all-cause mortality may include deaths unrelated to spondylodiscitis. All mortality analyses should therefore be regarded as exploratory.

## 5. Conclusions

In this small retrospective surgical cohort, the routine preoperative MRI report did not describe an epidural abscess in any of the 11 patients in whom epidural pus was later documented at surgery; paraspinal soft-tissue involvement had been reported in six of them. These observations concern routine reporting and clinical documentation rather than the diagnostic accuracy of MRI, and they warrant evaluation with blinded, protocol-based image review. In exploratory analyses, mortality was associated with age and the age-adjusted CCI, whose discrimination was similar, whereas the disease-specific classification systems were not associated with death. These findings are hypothesis-generating and require confirmation in larger, preferably multicentre, prospective cohorts.

## Figures and Tables

**Table 1:** Demographic and baseline clinical characteristics of the study population (*n* = 27).

Characteristic	Value
Age (years), median [IQR]	67 [60–70]
Age range (min–max)	28–77
Sex, *n* (%)	
Male	20 (74.1)
Female	7 (25.9)
ASA score, median [IQR]	3 [2–3]
Total length of index admission (days), median [IQR]	25 [22–29.5]
Orthopaedic ward	8 [4–13]
Intensive care unit	4 [2.5–5]
Infectious diseases ward	14 [3.5–18]
Follow-up duration (months), median [IQR]	2.5 [1.2–8.3]
Follow-up range (min–max)	0.3–32.5
Comorbidity profile	
Any comorbidity present, *n* (%)	23 (85.2)
Total number of comorbidities, median [IQR]	2 [1–3]
Cardiac comorbidity, *n* (%)	18 (66.7)
Metabolic comorbidity, *n* (%)	9 (33.3)
Renal comorbidity, *n* (%)	5 (18.5)
Immune comorbidity, *n* (%)	3 (11.1)
Pulmonary comorbidity, *n* (%)	3 (11.1)
Charlson Comorbidity Index, median [IQR]	1 [0–2]
Age-adjusted CCI, median [IQR]	3 [2–4]
Clinical outcomes, *n* (%)	
Neurological deficit at presentation	12 (44.4)
Neurological status at end of orthopaedic stay, patients with a deficit (resolved/improved/unchanged)	5/5/2
New deficit at end of orthopaedic stay, patients without a deficit	0 of 14 assessable (1 not assessable)
Reinfection during follow-up	4 (14.8)
Mortality during follow-up	11 (40.7)

IQR, interquartile range (25th–75th percentile); ASA, American Society of Anesthesiologists; CCI, Charlson Comorbidity Index. The age-adjusted CCI adds one point per decade over 40 years. Comorbidity categories are not mutually exclusive. Length of stay is given for the total index admission and for each ward; all patients spent time in intensive care, and 20 were transferred to the infectious diseases ward. Neurological status was graded with the ASIA Impairment Scale at the end of the orthopaedic ward stay: among the 12 patients presenting with a deficit, it had resolved in 5, improved in 5, and was unchanged in 2; of the 15 patients without a deficit at presentation, 14 had no new deficit and 1 could not be assessed.

**Table 2:** Microbiological, surgical, and radiological findings (*n* = 27).

Finding	*n*	%
Anatomical location of infection		
Thoracic	7	25.9
Thoracolumbar	4	14.8
Lumbar/lumbosacral	15	55.6
Multilevel/non-contiguous	1	3.7
Culture results		
Tissue culture positive	15	55.6
Blood culture positive	13	48.1
Microbiological profile		
Culture-negative (presumed pyogenic)	12	44.4
*Staphylococcus aureus (MSSA)*	7	25.9
*Staphylococcus aureus (MRSA)*	1	3.7
*Staphylococcus epidermidis*	2	7.4
*Acinetobacter baumannii*	1	3.7
*Pseudomonas* spp.	1	3.7
*Escherichia coli*	1	3.7
*Candida* spp.	1	3.7
*Mycoplasma hominis*	1	3.7
MRI findings		
Epidural abscess (named in report)	2	7.4
Cord compression	5	18.5
Vertebral body destruction	9	33.3
Endplate involvement	8	29.6
Paravertebral abscess	3	11.1
Intramedullary signal change	5	18.5
Paraspinal soft-tissue involvement	8	29.6
MRI severity composite score (0–6; exploratory), median [IQR]	1 [0–2]	
Surgical management		
Posterior approach	18	66.7
Anterior approach	3	11.1
Combined anterior–posterior approach	6	22.2
Direct epidural exploration and decompression	6	22.2
Spinal instrumentation performed	25	92.6
Instrumented levels, median [IQR]	4 [3–6]	
Epidural pus documented in operative note	11	40.7

IQR, interquartile range; MRI, magnetic resonance imaging. Microbiological categories are mutually exclusive. MRI findings were abstracted from routine radiology reports. The MRI severity composite score (0–6) is an exploratory, non-validated sum of six binary imaging features (epidural abscess, paravertebral abscess, cord compression, intramedullary signal change, vertebral body destruction, and endplate involvement); paraspinal soft-tissue involvement is reported separately and is not part of this composite. The epidural space was explored directly in 6 patients and inspected through the debridement corridor in the remaining 21. The comparison between MRI report findings and documented operative findings for epidural abscess is presented in Table 3.

**Table 3:** Epidural abscess in routine MRI reports versus documented operative findings (Panel A), discrimination for death during follow-up (Panel B), and univariable Cox models for all-cause mortality (Panel C) (*n* = 27).

Panel A. Findings in routine MRI reports versus epidural pus documented in operative notes.					
Finding in MRI report	Sensitivity	Specificity	PPV	NPV	McNemar *p*
Epidural abscess named in report	0% (0/11) [0–28.5]	87.5% (14/16) [61.7–98.4]	0% (0/2) [0–84.2]	56.0% (14/25) [34.9–75.6]	0.022
Paraspinal soft-tissue involvement reported	54.5% (6/11) [23.4–83.3]	87.5% (14/16) [61.7–98.4]	75.0% (6/8) [34.9–96.8]	73.7% (14/19) [48.8–90.9]	0.45
Panel B. Discrimination for death during follow-up (ROC analysis; secondary, descriptive).					
Measure		AUC		95% CI (DeLong)	
Age (years)		0.741		0.544–0.939	
Age-adjusted CCI		0.736		0.553–0.918	
CCI (age-unadjusted)		0.662		0.455–0.869	
MRI severity composite (exploratory)		0.625		0.405–0.845	
Pola classification		0.562		0.356–0.769	
SISS		0.531		0.345–0.717	
SINS		0.500		0.279–0.721	
Homagk classification		0.440		0.229–0.651	
Panel C. Univariable Cox proportional hazards models for all-cause mortality (primary mortality analysis).					
Measure		HR (95% CI); *p*		Harrell’s C	
Age (per year)		1.14 (1.00–1.30); 0.043		0.713	
Age-adjusted CCI (per point)		1.54 (1.05–2.28); 0.029		0.724	
CCI, age-unadjusted (per point)		1.45 (0.93–2.26); 0.098		0.665	
MRI severity composite (per point)		1.39 (0.93–2.09); 0.110		0.568	
Pola classification (per category)		1.30 (0.57–2.94); 0.533		0.491	
SISS (per point)		0.88 (0.49–1.59); 0.673		0.503	
SINS (per point)		1.00 (0.79–1.28); 0.978		0.392	
Homagk classification (per type)		0.92 (0.54–1.58); 0.767		0.460	

AUC, area under the receiver operating characteristic curve; CCI, Charlson Comorbidity Index; CI, confidence interval; HR, hazard ratio; MRI, magnetic resonance imaging; NPV, negative predictive value; PPV, positive predictive value; ROC, receiver operating characteristic; SINS, Spinal Instability Neoplastic Score; SISS, Spinal Instability Spondylodiscitis Score. Panel A: MRI findings were abstracted from routine clinical reports and compared with epidural pus documented in the operative notes; values are proportions with exact (Clopper–Pearson) 95% CIs in brackets; the exact McNemar test assessed directional discordance, and a non-significant result does not indicate agreement. For paraspinal soft-tissue involvement, the conditional odds ratio was 7.65 (exact 95% CI 0.97–102.2; Fisher exact *p* = 0.033). Panel B: AUCs for death during follow-up with DeLong 95% CIs; pairwise comparisons are given in Table S4. Panel C: univariable Cox models with follow-up time from surgery; Harrell’s C summarises discrimination. All mortality analyses are exploratory.

**Table 4:** Preoperative and postoperative day 7 laboratory parameters (Wilcoxon signed-rank test, *n* = 27).

Parameter	Preoperative, Median [IQR]	Postoperative Day 7, Median [IQR]	Z	*p*-Value
WBC (×10^3^/µL)	7.45 [5.94–9.92]	10.95 [9.21–13.13]	+3.41	0.001
Hemoglobin (g/dL)	10.40 [9.90–11.55]	10.00 [9.20–11.80]	−1.89	0.059
C-reactive protein (mg/L)	83.5 [35.0–228.5]	26.0 [15.4–70.5]	−2.83	0.005
ESR (mm/h)	66.0 [41.0–88.5]	32.0 [14.5–50.5]	−3.28	0.001
Albumin (g/dL)	3.20 [2.45–3.85]	2.63 [2.36–3.20]	−2.26	0.024

Values are presented as median [interquartile range]; postoperative values were obtained on postoperative day 7. The Wilcoxon signed-rank test was used to compare paired pre- and postoperative values; a positive Z denotes an increase and a negative Z a decrease from the preoperative value. IQR, interquartile range; WBC, white blood cell count; ESR, erythrocyte sedimentation rate.

## Data Availability

The datasets generated and analysed during the current study are not publicly available in order to protect patient privacy but are available from the corresponding author upon reasonable request.

## Supplementary Materials

The following supporting information can be downloaded at https://www.mdpi.com/article/10.3390/jcm15197665/s1. Table S1: Distribution of classification and severity scores; Table S2: Comparison of clinical variables between survivors and non-survivors; Table S3: Spearman correlation matrix among classification scores; Table S4: Pairwise DeLong comparisons of areas under the receiver operating characteristic curve for all-cause mortality.
