# Supplementary material for: Epidural Abscess in Routine Preoperative MRI Reports Versus Documented Operative Findings, and Exploratory Mortality Risk Factors in Surgically Treated Spondylodiscitis: A Retrospective Single-Centre Study
Source: J Clin Med. 2026 Oct 3;15(19):7665. doi: 10.3390/jcm15197665 (PMC13666127; doi:10.3390/jcm15197665)
Supplement: Supplementary file 1 [file jcm-15-07665-s001.zip › STROBE_STARD_Checklist.pdf]

STROBE Checklist — Cohort Studies

von Elm E et al. (2007), 22 items. The location column indicates where each item is addressed in the manuscript. Page and line numbers to be added after final typesetting.

Item	Recommendation topic	STROBE recommendation	Location in manuscript
Title and abstract			
1	Title & abstract	(a) Study design indicated in the title or abstract; (b) informative and balanced summary in the abstract	Title; Abstract (structured: Background/Methods/Results/Conclusions)
Introduction			
2	Background/rationale	Scientific background and rationale for the investigation	Introduction, paragraphs 1–4
3	Objectives	Specific objectives, including any prespecified hypotheses	Introduction, final paragraph (aims i–iii); Materials and Methods, "Study design and setting" (hypotheses)
Methods			
4	Study design	Key elements of study design early in the paper	Materials and Methods, "Study design and setting"
5	Setting	Setting, locations, and relevant dates	Materials and Methods, "Study design and setting" (2017–2025, single tertiary centre)
6	Participants	Eligibility criteria, sources, and methods of selection	Materials and Methods, "Cohort definition and eligibility"
7	Variables	Outcomes, exposures, predictors, potential confounders defined	Materials and Methods, "Clinical assessment and scoring systems", "Radiological assessment", "Follow-up and outcome definitions"
8	Data sources/measurement	Sources of data and details of methods of measurement	Materials and Methods, "Clinical assessment...", "Radiological assessment" (binary coding from routine reports)
9	Bias	Efforts to address potential sources of bias	Methods, Sections 2.3–2.4 (outcome-blinded consensus scoring; documented operative reference; report-based imaging data); Discussion, Section 4.7 (limitations)
10	Study size	How the study size was arrived at	Materials and Methods, "Cohort definition and eligibility" (consecutive patients; n = 27); Figure 1
11	Quantitative variables	How quantitative variables were handled	Materials and Methods, "Statistical analysis" (median/IQR; thresholds)
12	Statistical methods	All methods, subgroups, missing data, sensitivity analyses	Methods, Section 2.7 (Mann–Whitney U, Wilcoxon, Fisher, McNemar; exact Clopper–Pearson CIs; univariable Cox models; ROC with DeLong CIs and pairwise comparisons; Kaplan–Meier)
Results			
13	Participants	Numbers at each stage (eligible, included, analysed)	Results, "Cohort characteristics"; Figure 1 (STROBE flow diagram)
14	Descriptive data	Characteristics of participants and data for each variable	Results, "Cohort characteristics"; Table 1
15	Outcome data	Numbers of outcome events	Results (mortality 11/27); Table 1
16	Main results	Estimates, confidence intervals, adjustment for confounders	Results, Section 3.5; Table 3, Panels B–C (HR and AUC with 95% CI); no adjustment for confounders because of 11 events (Section 2.7)
17	Other analyses	Subgroup, interaction, and sensitivity analyses	Results (diagnostic performance; soft-tissue sub-analysis); Table 3, Supplementary Table S2
Discussion			
18	Key results	Summarise key results with reference to study objectives	Discussion (opening paragraph)
19	Limitations	Limitations, sources of bias and imprecision	Discussion (limitations paragraph)
20	Interpretation	Cautious overall interpretation in the context of evidence	Discussion (comparison with literature); Conclusions
21	Generalisability	External validity of the results	Discussion (single centre, n = 27, need for external validation)

Item	Recommendation topic	STROBE recommendation	Location in manuscript
Other information			
22	Funding	Source of funding and role of funders	Funding statement

STARD 2015 Checklist — Diagnostic-Accuracy Component

Bossuyt PM et al. (2015). This checklist applies to the component comparing routine MRI reports with documented operative findings for epidural abscess. Index test = epidural abscess named in the routine preoperative MRI report; reference standard = purulent material in the epidural space documented in the operative note.

Item	Section	STARD 2015 item	Location in manuscript
Title / Abstract / Keywords			
1	Title/Abstract	Identification as a study of diagnostic accuracy using at least one measure of accuracy	Title (“MRI Reports Versus Documented Operative Findings”); Abstract and Section 3.3 (sensitivity, specificity)
2	Abstract	Summary of design, methods, and results	Abstract (structured)
Introduction			
3	Introduction	Scientific and clinical background; intended use and clinical role of the index test; objectives	Introduction (agreement between routine MRI reports and operative findings); aim (i)
Methods			
4	Study design	Whether data collection was prospective or retrospective	Materials and Methods, "Study design and setting" (retrospective)
5	Participants	Eligibility criteria	Materials and Methods, "Cohort definition and eligibility"
6	Participants	Basis of participant identification (consecutive, random)	Materials and Methods (consecutive patients)
7	Participants	Setting, location, dates of identification	Materials and Methods, "Study design and setting"; "Radiological assessment" (routine clinical care)
8	Test methods	Index test, in sufficient detail to allow replication	Materials and Methods, "Radiological assessment" (1.5-T MRI; binary coding from routine reports)
9	Test methods	Reference standard, in sufficient detail to allow replication	Methods, Section 2.4 (purulent material in the epidural space documented in the operative note; inspection route described for every patient)
10	Test methods	Rationale for choosing the reference standard	Materials and Methods (direct surgical exploration as the closest anatomical reference)
11	Test methods	Definition and rationale for test positivity cut-offs / categories	Materials and Methods (binary "epidural abscess present/absent" coding)
12	Test methods	Whether readers of the index test were aware of reference-standard results	Materials and Methods + Discussion (findings from routine reports; blinded re-reading was not performed — stated as a limitation)
13	Test methods	Whether assessors of the reference standard were aware of index-test results	Materials and Methods (intraoperative findings from operative notes, documented separately)
14	Analysis	Methods for estimating or comparing measures of diagnostic accuracy	Materials and Methods, "Statistical analysis" (sensitivity, specificity, predictive values; McNemar exact test)
15	Analysis	How indeterminate results were handled	Materials and Methods (n = 27 complete data; incomplete records an exclusion criterion)
Results			
16	Participants	Flow of participants (eligible, included, analysed)	Figure 1 (STROBE flow diagram); Results
17	Participants	Baseline demographic and clinical characteristics	Results, "Cohort characteristics"; Table 1
18	Test results	Time interval and interventions between index test and reference standard	Methods, Sections 2.4–2.5 (MRI 1–7 days before surgery; no antibiotics before surgery)
19	Test results	Cross-tabulation of index-test by reference-standard results	Table 3, Panel A (TP/FP/FN/TN derivable)
20	Test results	Estimates of diagnostic accuracy and their precision (e.g., 95% CI)	Section 3.3; Table 3, Panel A (sensitivity, specificity, and predictive values with exact 95% CIs; McNemar p)

Item	Section	STARD 2015 item	Location in manuscript
21	Test results	Adverse events from index test or reference standard	Not applicable (non-interventional test; routine MRI and surgery)
Discussion			
22	Discussion	Study limitations, including bias and applicability	Discussion (limitations paragraph)
23	Discussion	Implications for practice; intended use and clinical role	Discussion (clinical implications)
Other information			
24	Registration	Registration number and name of registry	Not applicable (retrospective study)
25	Protocol	Where the full study protocol can be accessed	Not applicable (no separate protocol published)
26	Funding	Sources of funding and support; role of funders	Funding statement
