# Supplementary material for: Epidural Abscess in Routine Preoperative MRI Reports Versus Documented Operative Findings, and Exploratory Mortality Risk Factors in Surgically Treated Spondylodiscitis: A Retrospective Single-Centre Study
Source: J Clin Med. 2026 Oct 3;15(19):7665. doi: 10.3390/jcm15197665 (PMC13666127; doi:10.3390/jcm15197665)
Supplement: Supplementary file 1 [file jcm-15-07665-s001.zip › Supplementary_Table_S1.pdf]

Supplementary Table S1. Distribution of classification and severity scores (N = 27)

Score	Mean ± SD	Median [IQR]	Category distribution, n (%)
Pola classification	—	—	A: 4 (14.8); B: 12 (44.4); C: 11 (40.7)
Homagk classification	2.78 ± 1.05	3 [2–3]	Type 1: 2 (7.4); Type 2: 11 (40.7); Type 3: 6 (22.2); Type 4: 7 (25.9); Type 5: 1 (3.7)
SINS	7.89 ± 2.14	9 [7–9]	Stable: 4 (14.8); Potentially unstable: 23 (85.2); Unstable: 0 (0.0)
SISS	4.19 ± 1.11	4 [4–4]	Intermediate: 26 (96.3); Unstable: 1 (3.7)
CCI	1.30 ± 1.35	1 [0–2]	Low (≤2): 20 (74.1); High (>2): 7 (25.9)
Age-adjusted CCI	3.26 ± 1.79	3 [2–4]	Low (≤3): 17 (63.0); High (>3): 10 (37.0)
MRI severity score (0–6)	1.19 ± 1.24	1 [0–2]	0: 9 (33.3); 1: 9 (33.3); 2: 7 (25.9); 4: 1 (3.7); 5: 1 (3.7)

SD, standard deviation; IQR, interquartile range; SINS, Spinal Instability Neoplastic Score; SISS, Spinal Instability Spondylodiscitis Score; CCI, Charlson Comorbidity Index; MRI, magnetic resonance imaging. The age-adjusted CCI adds one point per decade over 40 years. The MRI severity composite score (range 0–6) integrates six binary imaging features (epidural abscess, paravertebral abscess, cord compression, cord signal change, vertebral destruction, endplate involvement). Data represent the final analytical cohort (N = 27) following a priori exclusion of tuberculous cases. The SISS score shows very low variability in this cohort, with 96.3% of patients scoring within the intermediate range.
