# Supplementary material for: Epidural Abscess in Routine Preoperative MRI Reports Versus Documented Operative Findings, and Exploratory Mortality Risk Factors in Surgically Treated Spondylodiscitis: A Retrospective Single-Centre Study
Source: J Clin Med. 2026 Oct 3;15(19):7665. doi: 10.3390/jcm15197665 (PMC13666127; doi:10.3390/jcm15197665)
Supplement: Supplementary file 1 [file jcm-15-07665-s001.zip › Supplementary_Table_S2.pdf]

Supplementary Table S2. Comparison of clinical variables between survivors and non-survivors (N = 27)

Variable	Survivors (n = 16)	Non-survivors (n = 11)	Test	p-value
Continuous variables (mean rank)				
Age (years)	11.34	17.86	U = 45.5	0.038
Age-adjusted CCI	11.41	17.77	U = 46.5	0.037
Charlson Comorbidity Index	12.22	16.59	U = 59.5	0.149
MRI severity score	12.62	16.00	U = 66.0	0.266
SINS	14.00	14.00	U = 88.0	1.000
Homagk classification	14.66	13.05	U = 77.5	0.604
SISS	13.66	14.50	U = 82.5	0.768
Categorical variables, n (%)				
Preoperative neurological deficit	6 (37.5)	6 (54.5)	Fisher	0.452
Tissue culture positive	8 (50.0)	7 (63.6)	Fisher	0.696
Blood culture positive	6 (37.5)	7 (63.6)	$\chi^2 = 1.78$	0.182
Pola class C	6 (37.5)	5 (45.5)	Fisher	0.710

CCI, Charlson Comorbidity Index; MRI, magnetic resonance imaging; SINS, Spinal Instability Neoplastic Score; SISS, Spinal Instability Spondylodiscitis Score. Continuous variables were compared with the Mann–Whitney U test (mean ranks shown); categorical variables with the chi-square (χ^2) or Fisher exact test. Data represent the final analytical cohort (N = 27) following a priori exclusion of tuberculous cases. Only age and the age-adjusted CCI differed significantly between survivors and non-survivors.
