# Supplementary material for: Epidural Abscess in Routine Preoperative MRI Reports Versus Documented Operative Findings, and Exploratory Mortality Risk Factors in Surgically Treated Spondylodiscitis: A Retrospective Single-Centre Study
Source: J Clin Med. 2026 Oct 3;15(19):7665. doi: 10.3390/jcm15197665 (PMC13666127; doi:10.3390/jcm15197665)
Supplement: Supplementary file 1 [file jcm-15-07665-s001.zip › Supplementary_Table_S3.pdf]

Supplementary Table S3. Spearman correlation matrix among classification scores (N = 27).

	Pola	Homagk	SINS	SISS
Pola	—	0.585 (p = 0.001)	0.671 (p < 0.001)	0.458 (p = 0.016)
Homagk		—	0.411 (p = 0.033)	0.115 (p = 0.569)
SINS			—	0.254 (p = 0.201)
SISS				—

Values are Spearman rank correlation coefficients (ρ) with two-sided p-values, computed with IBM SPSS Statistics version 27. Interpretation: $|\rho| < 0.30$ weak, $0.30 \le |\rho| < 0.60$ moderate, $|\rho| \ge 0.60$ strong. Pola correlated moderately to strongly with Homagk, SINS, and SISS; Homagk correlated moderately with SINS but not with SISS; SINS and SISS were not significantly correlated. SINS, Spinal Instability Neoplastic Score; SISS, Spinal Instability Spondylodiscitis Score.
