# Supplementary material for: Epidural Abscess in Routine Preoperative MRI Reports Versus Documented Operative Findings, and Exploratory Mortality Risk Factors in Surgically Treated Spondylodiscitis: A Retrospective Single-Centre Study
Source: J Clin Med. 2026 Oct 3;15(19):7665. doi: 10.3390/jcm15197665 (PMC13666127; doi:10.3390/jcm15197665)
Supplement: Supplementary file 1 [file jcm-15-07665-s001.zip › Supplementary_Table_S4.pdf]

Supplementary Table S4. Pairwise DeLong comparisons of areas under the receiver operating characteristic curve for death during follow-up (N = 27).

Comparison	ΔAUC (95% CI)	p
Age-adjusted CCI vs. age	−0.006 (−0.202 to 0.190)	0.955
Age-adjusted CCI vs. CCI	0.074 (−0.056 to 0.203)	0.263
Age vs. CCI	0.080 (−0.209 to 0.368)	0.588
Age-adjusted CCI vs. MRI severity composite	0.111 (−0.195 to 0.417)	0.478
Age-adjusted CCI vs. Pola	0.173 (−0.098 to 0.445)	0.211
Age-adjusted CCI vs. Homagk	0.295 (0.019 to 0.572)	0.036
Age-adjusted CCI vs. SINS	0.236 (−0.060 to 0.531)	0.118
Age-adjusted CCI vs. SISS	0.205 (−0.056 to 0.465)	0.124

ΔAUC is the difference in area under the curve (first minus second measure) with DeLong 95% confidence intervals and p-values, computed in Python (see Methods). No adjustment was made for multiple comparisons; with a Bonferroni correction for eight comparisons ($\alpha = 0.00625$), none of the differences would be significant. AUC, area under the receiver operating characteristic curve; CCI, Charlson Comorbidity Index; CI, confidence interval; MRI, magnetic resonance imaging; SINS, Spinal Instability Neoplastic Score; SISS, Spinal Instability Spondylodiscitis Score.
